# Research on Object Detection and Tracking Methods for aLow-Speed Mobile Platform

**DOI:** 10.3390/s25226869

**Published:** 2025-11-10

**Authors:** Gang Liu, Tao Jiang, Ming Ye, Yang Xu, Pengyu Zhao

**Affiliations:** 1 Automotive Parts Advanced Manufacturing Technology Education Key Laboratory, Chongqing University of Technology, Chongqing 400054, China; 2 Chongqing Zhuang Zhou Technology Co., Ltd., Chongqing 401120, China

**Keywords:** multi-sensor fusion, Ultra-Wideband (UWB), gait feature recognition, Extended Kalman Filter (EKF), trajectory tracking, Model Predictive Control (MPC)

## Abstract

Enhancing the positioning stability and accuracy of autonomous following systems poses a significant challenge, particularly in dynamic indoor environments susceptible to occlusion and interference. This paper proposes an innovative approach that integrates Ultra-Wideband (UWB) technology with computer vision-based gait analysis to overcome these limitations. First, a low-power, high-update-rate UWB positioning network is established based on an optimized Double-Sided Two-Way Ranging (DS-TWR) protocol. To compensate for UWB’s deficiencies under Non-Line-of-Sight (NLOS) conditions, a visual gait recognition process utilizing the GaitPart framework is introduced for target identification and relative motion estimation. Subsequently, an Extended Kalman Filter (EKF) is developed to seamlessly fuse absolute UWB measurements with gait-based relative kinematic information, thereby generating precise and robust estimates of the leader’s trajectory. This estimated path is tracked by a differentially driven mobile platform via a Model Predictive Controller (MPC). Experimental results demonstrate that the tracking deviation for most trajectory points remains within 50 mm, with a maximum observed deviation of 115 mm during turns, confirming its strong robustness and practical utility in real-world intelligent vehicle applications.

## 1. Introduction

In recent years, with the rapid advancement of intelligent sensing and robotics technologies, smart mobile platforms have been progressively permeating daily applications such as logistics handling, medical transport, and retail warehousing, aiming to enhance operational efficiency and reduce labor costs [1,2,3]. Among these applications, low-speed mobile platforms (e.g., shopping carts, luggage carriers, and flatbed trolleys) have become ideal research subjects for autonomous following technologies due to their widespread applicability. The core challenge in achieving reliable autonomous following lies in the stable and accurate positioning of the human leader and the understanding of their behavioral intentions.

The current mainstream tracking technologies primarily rely on visual sensing or wireless radio frequency (RF) positioning. Visual-based approaches, such as feature point tracking [4] or visual servoing [5], are widely used in SLAM systems [6]. These methods provide rich perceptual information and effectively support real-time localization and map building in ideal environments with good lighting and no occlusions. In this context, probabilistic robotics plays a crucial role as a powerful and mainstream solution. By utilizing probabilistic models to handle uncertainty within the system, it significantly enhances the robustness and accuracy of SLAM systems. However, visual approaches are highly sensitive to sudden lighting changes, background interference, and occlusion of the target [7], limiting their robustness in complex indoor environments, which, in turn, challenges the accuracy and stability of the SLAM system. On the other hand, positioning solutions based on wireless technologies such as Bluetooth, Wi-Fi, and ZigBee face issues like limited accuracy and poor resistance to multipath interference, making it difficult to meet the high-precision tracking requirements [8,9,10].

Given that the target environment is indoors, common issues include long corridors, repetitive structures, and dim lighting. Many advanced SLAM solutions rely on environmental features, which can be disrupted under such conditions. RF trilateration, however, offers strong penetration and is less affected by the aforementioned challenges in visual systems. Additionally, RF signal is relatively low-cost and energy-efficient.

Ultra-Wideband (UWB) technology has demonstrated significant advantages in indoor positioning owing to its high-precision ranging capability, strong anti-interference performance, and excellent time resolution [11,12]. It provides mobile platforms with stable and absolute positional references, effectively compensating for the limitations of vision-only solutions. However, UWB technology has inherent limitations: signals are prone to interruption under Non-Line-of-Sight (NLoS) conditions, and target confusion (i.e., the inability to distinguish specific individuals) can occur in multi-target scenarios, leading to following errors [13].

To address these challenges, multi-modal sensor fusion has become an essential trend for enhancing system robustness. In particular, gait features serve as a unique biometric identifier that enables identity recognition and motion intention prediction by analyzing periodic spatiotemporal changes in walking patterns [14,15]. This capability effectively resolves UWB’s target confusion issue and provides continuous motion estimation during temporary UWB signal loss. Although prior studies have explored the use of UWB for robot tracking [16] or the fusion of UWB with IMUs [17], most of these works have not adequately addressed target identification and dynamic occlusion in complex indoor environments. Furthermore, there is a lack of systematic research and validation on the deep integration of UWB and visual gait analysis in realistic indoor scenarios that involve turning motions.

To address the aforementioned challenges, this paper investigates an autonomous following system for low-speed mobile platforms by fusing UWB positioning with gait characteristics. The main contributions are threefold: (1) It presents a novel deep fusion framework integrating UWB and gait information, effectively addressing the challenge of continuous target tracking under occlusion conditions. (2) It designs a tracking controller based on an Extended Kalman Filter (EKF) for trajectory estimation and Model Predictive Control (MPC), achieving centimeter-level accurate tracking in indoor environments. (3) It accomplishes full-stack system integration and empirical validation on a typical low-speed mobile platform.

## 2. Problem Modeling and System Framework

This research aims to address the problem of robust autonomous tracking of a specific leader by a low-speed mobile platform in dynamic indoor environments. The core of this problem can be decomposed into two key sub-problems: Leader Trajectory Estimation: how to fuse UWB absolute positioning information with visual gait-based relative motion information to generate a smooth, continuous, and accurate estimation of the leader’s trajectory; and Trajectory Tracking Control: how to design a controller based on the estimated leader trajectory to drive a differentially driven mobile platform subject to nonholonomic constraints, enabling precise and stable tracking while adhering to its own kinematic constraints.

**Figure 1:**
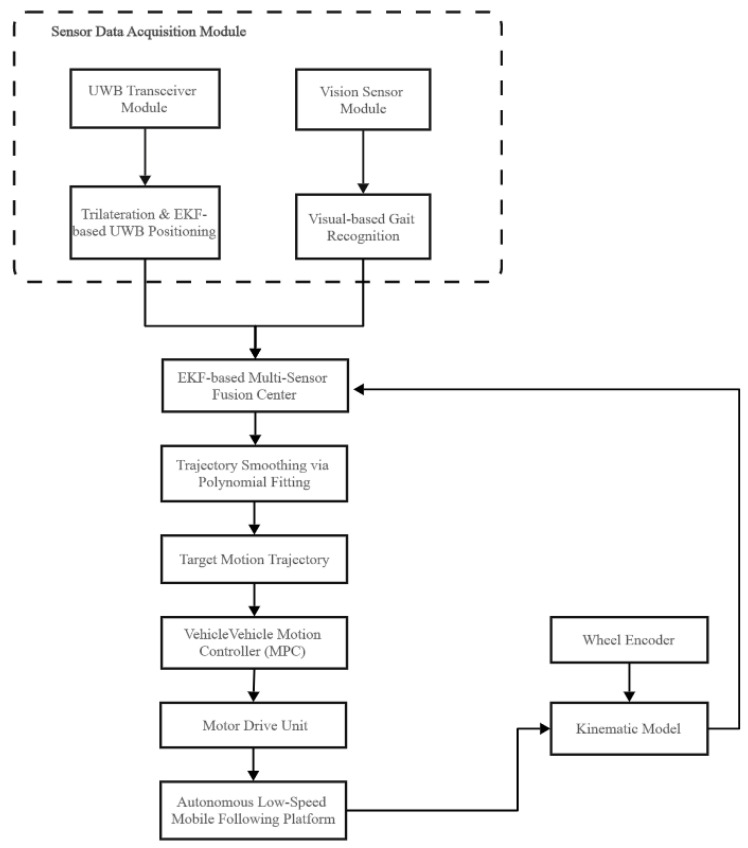
System solutions.

To this end, this paper designs an overall system technical framework (as shown in Figure 1). This architecture adheres to the classic perception–planning–execution paradigm in robotics and integrates core modules such as Ultra-Wideband (UWB) positioning, visual gait recognition, multi-sensor filtering and fusion, and Model Predictive Control (MPC) to achieve robust autonomous tracking in dynamic indoor environments. The system workflow is as follows: at the perception layer, the UWB positioning module and the visual sensor synchronously collect raw distance and image data of the leader; at the control layer, the onboard computer (Raspberry Pi) is responsible for fusing multi-source sensor information, estimating the leader’s trajectory, and generating motion control commands; at the execution layer, the lower-level controller (STM32) executes the control commands with high-frequency closed-loop control of the motor drivers. The entire system is powered by a dedicated power supply module optimized for long-endurance operation of the mobile platform.

## 3. UWB-Based Leader Localization Method

This chapter elaborates on the UWB-based leader positioning method, covering the ranging principle, multi-base station timing design, data filtering processing, and the final coordinate calculation via trilateral positioning.

### 3.1. Principles and Methods of UWB Ranging

**Figure 2:**
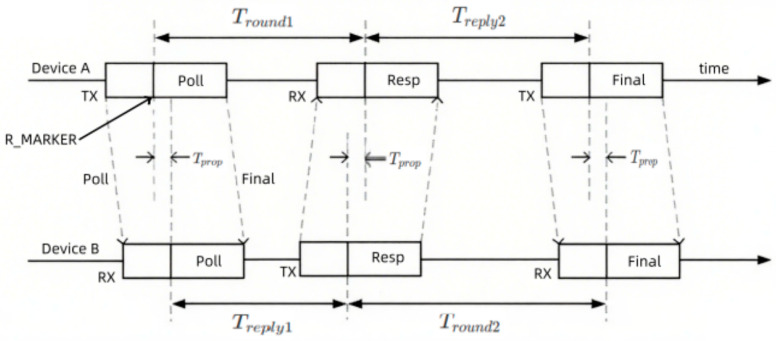
DS-TWR schematic diagram.

Accurate ranging serves as the foundation of UWB positioning. This study employs a ranging method based on Time of Flight (ToF), specifically the Double-Sided Two-Way Ranging (DS-TWR) protocol. This method achieves high-precision distance measurement without requiring strict clock synchronization by utilizing multiple signal exchanges. Its fundamental principle [18] is illustrated in Figure 2.

Due to clock asynchronization, the time measurements obtained by the devices contain biases. Let the deviation coefficient of the tag be K_A_, and that of the base station be K_B_. Due to clock asynchronization, the time measured by the devices contains deviations. The relationship between the values T′_round1_ and T′_reply2_ measured by the tag and their true values is as follows:

$${T^{\prime }}_{round1}=K_{A}T_{round1},{T^{\prime }}_{reply2}=K_{A}T_{reply2}$$

Similarly, the time measured by the base station follows an analogous relationship:

$${T^{\prime }}_{round2}=K_{B}T_{round2},{T^{\prime }}_{reply1}=K_{B}T_{reply1}$$

DS-TWR calculates the flight time T_prop_ using the following formula:

(1) $$T_{prop}=\frac{{T^{\prime }}_{round1}\cdot {T^{\prime }}_{round2}-{T^{\prime }}_{reply1}\cdot {T^{\prime }}_{reply2}}{{T^{\prime }}_{round1}+{T^{\prime }}_{round2}+{T^{\prime }}_{reply1}+{T^{\prime }}_{reply2}}$$

Here, T_prop_ represents the signal flight time between the master node and the target node. Substituting the biased measured values into the above formula yields. Assuming K_A_ ≈ K_B_ = K (all devices are from the same batch, with deviations in the same direction and of similar magnitude), the formula can be simplified to:

(2) $$T_{prop}\approx K\frac{\left({T^{\prime }}_{round1}\cdot {T^{\prime }}_{round2}-{T^{\prime }}_{reply1}\cdot {T^{\prime }}_{reply2}\right)}{\left({T^{\prime }}_{round1}+{T^{\prime }}_{round2}+{T^{\prime }}_{reply1}+{T^{\prime }}_{reply2}\right)}$$

Theoretical analysis demonstrates that this formula effectively eliminates the primary error term arising from the clock frequency difference between the tag and the base station. Taking the typical 20 ppm accuracy crystal oscillator employed in our system as an example, the maximum ranging error caused by the residual scale error is merely on the millimeter level within a 50 m range, which is significantly smaller than the impact of channel noise. Therefore, DS-TWR can provide reliable distance observations for the system.

### 3.2. Ranging Time Sequence in Multi-Base Station Mode

**Figure 3:**
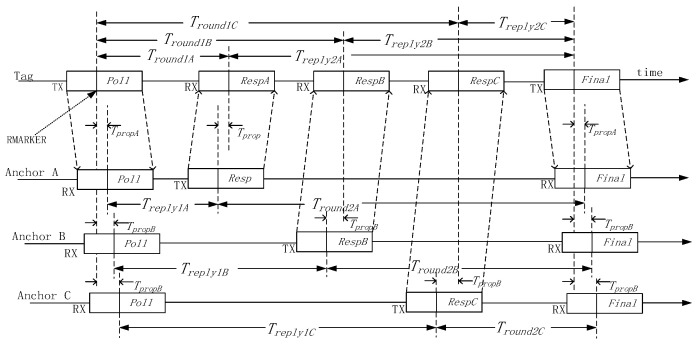
Base station positioning sequence diagram.

To reduce the power consumption of the tag in multi-base station scenarios, this paper improves the traditional ranging sequence. As shown in Figure 3, the tag broadcasts a Poll message, which can be received by all base stations. After the base stations sequentially reply with Resp messages, the tag broadcasts a Final message. This design reduces the number of transmissions required from the tag from 6 times (for three base stations) to only 2 times, significantly lowering communication overhead and power consumption.

Taking Anchor A as an example, the Time of Flight (TOF) of the signal from the tag to Anchor A is denoted as $T_{TOFA}$.

(3) $$T_{TOFA}=\frac{T_{round1A}\cdot T_{round2A}-T_{reply1A}\cdot T_{reply2A}}{T_{round1A}+T_{round2A}+T_{reply1A}+T_{reply2A}}$$

This time can be calculated using its measured values $T_{reply1A}$ and $T_{round2A},T_{reply2A}$ and $T_{round1A}$ transmitted back by the tag via the Final message. The same principle applies to Base Stations B and C.

The endurance test results (presented in Table 1 and Table 2) indicate that:

The tag power consumption does not increase significantly with the rise in the number of base stations, and the battery life remains stable.

### 3.3. Low-Pass Filtering Processing for UWB Ranging Data

To suppress random ranging noise caused by environmental electromagnetic interference and multipath effects, this paper employs a first-order low-pass filter to smooth the raw UWB ranging data. This filter features low computational complexity and excellent real-time performance. Its discrete form is given by:

(4) $${\hat{d}}_{k}=\alpha d_{k}+(1-\alpha ){\hat{d}}_{k-1}$$

where d_k_ represents the raw UWB ranging value at time step k, ${\hat{d}}_{k}$ denotes the filtered estimate, and α is the filter coefficient (0 < α ≤ 1). Based on an analysis of the system’s real-time requirements and noise characteristics, the optimal value of α is determined to be 0.4 in this study.

### 3.4. Trilateral Positioning to Calculate Leader Coordinates

**Figure 4:**
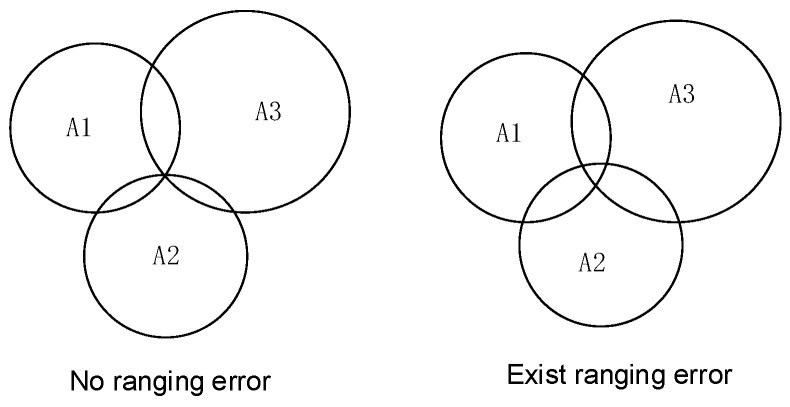
Three-sided positioning. A1, A2, and A3 represent the three anchor nodes with known coordinates; P denotes the target node to be located; the dashed circles indicate the communication ranges of the anchor nodes.

**Figure 5:**
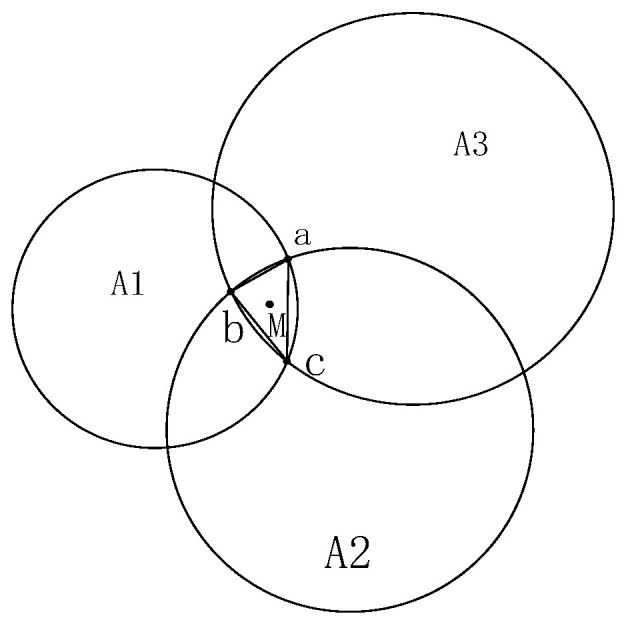
Centroid algorithm based on trilateral positioning. (a) Initial estimation of the target’s position using trilateration. (b) Formation of the centroid polygon by connecting the intersection points of the ranging circles. (c) Calculation of the final position as the centroid of the formed polygon.

To acquire the two-dimensional position coordinates of the navigator, the classic trilateration method [19] is used to calculate the initial coordinates of the tag. This method is straightforward and effective, helping to mitigate the impact of random errors. Ideally, the three circles should intersect at a single point. However, due to ranging errors, an overlapping region typically forms (as shown in Figure 4). To address this, this paper employs the centroid method to determine the final position: the points a, b, and c, formed by the pairwise intersections of the three circles, constitute a triangle, and their centroid is taken as the positioning result (Figure 5). The calculation formula is as follows:

(5) $$\left\{\begin{array}{c} x_{M}=\frac{x_{a}+x_{b}+x_{c}}{3}\\ y_{M}=\frac{y_{a}+y_{b}+y_{c}}{3} \end{array}\right.$$

## 4. Vision-Based Gait Feature Recognition Method

In order to maintain the tracking capability of the system in scenarios where UWB performance degrades, such as visual occlusion, this chapter introduces vision-based gait recognition as a complementary perception module.

### 4.1. YOLOv5 Object Detection Fused with UWB Positioning Information

**Figure 6:**
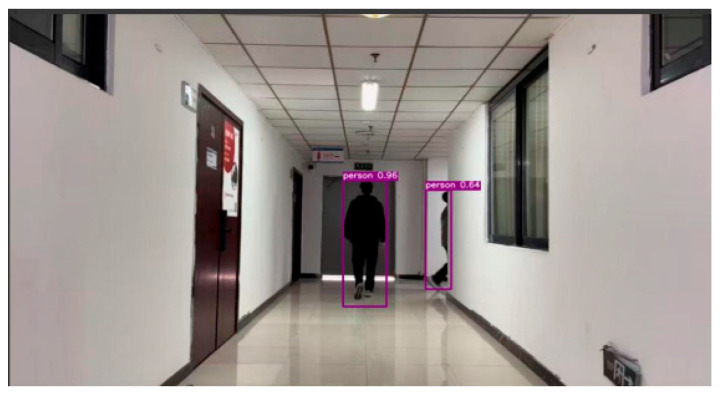
YOLOv5 Object Detection.

To enhance the robustness of target detection under occlusion conditions, this paper utilizes the coarse target position information provided by UWB to assist visual detection. The specific procedure involves mapping the absolute target coordinates obtained from UWB solution to the image coordinate system via perspective transformation, thereby deriving an estimated pixel region of the target. This region is used to construct a Candidate Box Optimization Region (CBOR). The confidence scores of YOLOv5 detection boxes falling within the CBOR are subsequently enhanced, as shown in Figure 6,which reduces the risk of correct targets being mistakenly eliminated during the Non-Maximum Suppression (NMS) stage due to occlusion.

To ensure temporal synchronization between the UWB positioning data and the image frames during the detection process, it is necessary to address the issues caused by different data acquisition frequencies and inconsistent timestamps. A nearest-neighbor matching method is introduced to align UWB data with image frames by minimizing the time difference. Let $t_{i}^{img}$ denote the timestamp of the i-th image frame, and represent the set of UWB data timestamps.

(6) $$T=\left\{t_{1,\;}t_{2,}...t_{n}\right\}$$

The corresponding *UWB* data can be selected using the following criterion:

(7) $$t_{UWB}=arg\underset{t_{j}\in T_{\mathrm{UWB}}}{\min}\left|t_{j}-t_{i}^{img}\right|$$

That is, the *UWB* data with the closest timestamp to $t_{i}^{img}$ is chosen, ensuring synchronization between the *UWB* information and the image frame.

### 4.2. Target Contour Extraction

**Figure 7:**
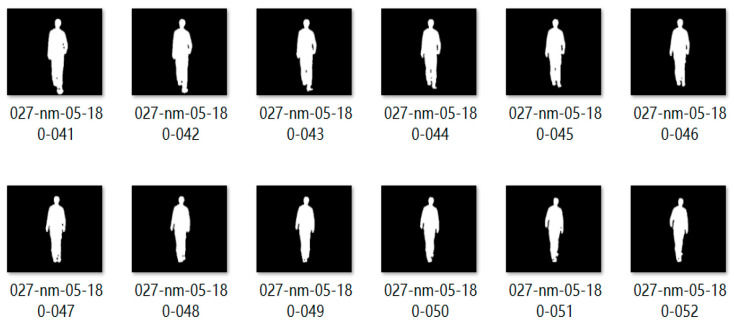
Sample standardized target contour images.

After the target is detected, the human silhouette is extracted based on the background subtraction method [20]. Following threshold segmentation and morphological closing operations, batch normalization is applied. The centroid is calculated and used as the central axis to standardize the contour image into a target silhouette map of size 64 × 44 pixels (Figure 7).

### 4.3. Gait Feature Extraction Based on GaitPart

Based on the standardized gait contour sequences extracted previously, this study employs the GaitPart network [21] for gait feature extraction. Let the input be a preprocessed gait contour image sequence I = {I_1_, I_2_, …, I_t_}, where I_t_∈R^64^ × ^44^ represents the binarized contour image of the t-th frame, and T is the sequence length. The specific implementation includes the following steps:

Each contour image I_t_ is uniformly divided into P local regions {R_t__1_, R_t__2_, …, R_tp_}. This paper adopts a division strategy of P = 8, where each region corresponds to a key motion part of the human body to capture local motion patterns.

For each local region *R_tp_*, features are extracted via a Frame-level Part Feature Extractor (FPFE):

(8) $$f_{t}^{p}=\;\mathrm{FPFE}(R_{t}^{p},\theta_{FPFE})$$

where $f_{t}^{p}$∈R^C^ is the C-dimensional feature vector, and *θ_FPFE_* denotes the network parameters of the FPFE. This paper employs ResNet-18 as the backbone network, with the feature dimension C set to 256.

To effectively integrate temporal information, the temporal feature sequence {$f_{1}^{p}$, $f_{2}^{p}$, …,$f_{t}^{p}$} of each local part is aggregated using Temporal Pyramid Aggregation (TPA) to obtain its corresponding global feature *g*. The features from all local parts are then concatenated to form the global gait feature vector:

(9) $$g=[h^{1},\;h^{2},\;...,h^{p}]\in R^{\mathrm{c.}}$$

Based on the aforementioned parameter configuration (P = 8, C = 256), a final d = 2048-dimensional global feature vector g is obtained.

The GaitPart network was implemented in PyTorch (Version 1.8.1), initialized with weights pre-trained on the CASIA-B dataset, and underwent lightweight modifications tailored for deployment on mobile platforms. The model was trained for 50 epochs to ensure full convergence of the loss function. The Adam optimizer was employed with a learning rate of 1 × 10^−4^ and momentum parameters *β_1_* = 0.9 and *β_2_* = 0.999.

Utilizing a step decay schedule. The ArcFace loss function, with a scale factor set to 32 and a margin parameter of 0.1, was adopted to enhance feature discriminability. To validate the advantages of the proposed method, comparative experiments were conducted on the CASIA-B dataset against traditional methods, including GEI + SVM [22] and HOG + RNN [23]. Table 3 presents the performance comparison of these methods under three different testing conditions:

Different levels of Gaussian noise are added to the contour images (as shown in Table 4).

The proposed method achieved the highest recognition rates under all three conditions—normal walking (NM), carrying a bag (BG), and wearing a coat (CL)—with an average accuracy of 90.5%. Furthermore, it exhibited the smallest performance degradation in robustness tests with added Gaussian noise, demonstrating its superiority.

### 4.4. Following Path Generation by Fusing Localization and Recognition

The aforementioned UWB positioning and visual gait recognition each possess distinct advantages and limitations: UWB provides absolute positioning but is susceptible to NLOS conditions, while gait offers continuous relative motion but suffers from cumulative error. To overcome these limitations inherent to individual sensors, this chapter designs a fusion algorithm based on the Extended Kalman Filter (EKF) to generate smooth, continuous, and accurate trajectory estimates of the leader.

To accurately characterize the relative motion relationship between the leader and the mobile platform, a world coordinate system is defined with its origin at the initial position of the mobile platform upon system activation. A mobile platform coordinate system is also established, which moves along with the platform and has its x-axis aligned with the platform’s forward direction.

The state vector is defined in this study as the motion state of the leader within the mobile platform coordinate system:

(10) $$x_{k}={\left[p_{x},p_{y},v_{x},v_{y}\right]}_{k}^{T}$$

where *p_x_* and *p_y_* represent the two-dimensional position coordinates of the leader, and *v_x_* and *v_y_* denote its translational velocities along the *x* and *y* directions, respectively.

Considering the continuity of human walking motion, the leader is assumed to move with constant velocity during adjacent sampling intervals (Δ*t* = 0.1 s). The corresponding linear state-space model is formulated as follows:

(11) $$x_{k}=Fx_{k-1}+w_{k}$$

where the state transition matrix is:

(12) $$F=\left[\begin{array}{cccc} 1 & 0 & \Delta t & 0\\ 0 & 1 & 0 & \Delta t\\ 0 & 0 & 1 & 0\\ 0 & 0 & 0 & 1 \end{array}\right]$$

The process noise wk~*N*(0, *Q*) characterizes the discrepancy between the constant velocity model and actual motion (acceleration/deceleration). With reference to the continuous-time white noise acceleration model, the process noise covariance matrix *Q* is derived through discretization based on biomechanical studies where the standard deviation of normal human walking acceleration is approximately 0.2 m/s^2^ [24]:

(13) $$Q=q\left[\begin{array}{cccc} \frac{{\Delta t}^{4}}{4} & 0 & \frac{{\Delta t}^{2}}{2} & 0\\ 0 & \frac{{\Delta t}^{2}}{2} & 0 & \frac{{\Delta t}^{4}}{4}\\ \frac{{\Delta t}^{2}}{2} & 0 & \Delta t^{2} & 0\\ 0 & \frac{{\Delta t}^{2}}{2} & 0 & \Delta t^{2} \end{array}\right]$$

Here, *q* is the process noise intensity parameter.

The system is equipped with two observational sensors.

UWB Observation Model: The *UWB* sensor provides polar coordinate observations of the navigator relative to the mobile platform:

(14) $$z_{k}^{UWB}={\left[d,\theta \right]}_{k}^{T}$$

where *F* denotes the state transition matrix, Δ*t* is the sampling time interval, and *w_k_* represents the process noise, which follows a Gaussian distribution with zero mean and covariance matrix *Q*. Observations in the system originate from two sensors. UWB observations provide the absolute distance (*d*) and azimuth angle (*θ*) of the leader.

(15) $$h^{UWB}(x_{k})=\left[\begin{array}{c} \sqrt{p_{x}^{2}+p_{y}^{2}}\\ arctan2(p_{y},p_{x}) \end{array}\right]$$

*UWB* Observation Noise $v_{k}^{UWB}$~*N*(0, *R^UWB^*):

(16) $$R^{UWB}=\left[\begin{array}{cc} \sigma_{d}^{2} & 0\\ 0 & \sigma_{\theta }^{2} \end{array}\right]$$

Based on the technical manual of the adopted DW1000 UWB module, the standard deviations of the observation noise were set as σ_d_ = 0.1 m and σ_θ_ = 5°(0.087 rad).

Gait Observation Model: Based on the gait recognition results, the relative position increment of the navigator in the platform coordinate system can be directly obtained, and its observation equation is linear:

(17) $$z_{k}^{Gait}=h^{Gait}(x_{k})+v_{k}^{Gait}=\left[\begin{array}{c} p_{x}\\ p_{y} \end{array}\right]+v_{k}^{Gait}$$

Here, $v_{k}^{UWB}$ and $v_{k}^{Gait}$ represent the observation noises, which follow zero-mean Gaussian distributions with covariance matrices $R_{UWB},R_{Gait}$, respectively. Since the gait observation model is linear, its observation matrix is given by ${\;H}_{Gait}=\left[\begin{array}{cc} 1 & \begin{array}{ccc} 0 & 0 & 0 \end{array}\\ 0 & \begin{array}{ccc} 1 & 0 & 0 \end{array} \end{array}\right]\Delta t$.

The gait observation noise $v_{k}^{Gait}$~*N*(0,$R^{Gait}$) is modeled based on the error propagation law and the Cramér–Rao Lower Bound analysis, incorporating the representational capacity of the GaitPart network. Accordingly, a diagonal covariance matrix is constructed as *R^Gait^* = diag(0.0025, 0.0025).

The EKF recursively fuses data through prediction and update steps. The prediction step projects the prior state and covariance based on the motion model. The update step is then performed depending on the availability of valid sensor data.

Prediction Step:

(18) $${\hat{x}}_{k|k-1}=F{\hat{x}}_{k-1|k-1}$$

(19) $$P_{k|k-1}=F{\hat{x}}_{k-1|k-1}F^{T}+Q$$

Here, ${\hat{x}}_{k|k-1}$ denotes the predicted state estimate, and $P_{k|k-1}$ represents the predicted state covariance matrix.

Update Step:

When UWB data is available, the Jacobian matrix $H_{k}^{UWB}$ of the UWB observation model is computed as follows:

(20) $$H_{k}^{UWB}={\frac{{\partial h}^{UWB}}{\partial x}|}_{{\hat{x}}_{k|k-1}}=\left[\begin{array}{cc} \frac{p_{x}}{\sqrt{p_{x}^{2}+p_{y}^{2}}} & \begin{array}{ccc} \frac{p_{y}}{\sqrt{p_{x}^{2}+p_{y}^{2}}} & 0 & 0 \end{array}\\ -\frac{y}{p_{x}^{2}+p_{x}^{2}} & \begin{array}{ccc} \frac{x}{p_{x}^{2}+p_{x}^{2}} & 0 & 0 \end{array} \end{array}\right]$$

Subsequently, the Kalman gain $K_{k}$ is computed, and the state and covariance are updated as follows:

(21) $$K_{k}=P_{k|k-1}{\left(H_{k}^{UWB}\right)}^{T}{\left(H_{k}^{UWB}P_{k|k-1}{\left(H_{k}^{UWB}\right)}^{T}+R^{UWB}\right)}^{-1}$$

(22) $${\hat{x}}_{k|k}={\hat{x}}_{k|k-1}+K_{k}(z_{k}^{UWB}-h^{UWB}{\hat{x}}_{k|k-1})$$

(23) $$P_{k|k}=(I-K_{k}H_{k}^{UWB})P_{k|k-1}$$

When *UWB* data is unavailable, the state and covariance are updated using only the gait observation:

(24) $$K_{k}=P_{k|k-1}{\left(H^{Gait}\right)}^{T}{\left(H^{Gait}P_{k|k-1}{\left(H^{Gait}\right)}^{T}+R^{Gait}\right)}^{-1}$$

(25) $${\hat{x}}_{k|k}={\hat{x}}_{k|k-1}+K_{k}(z_{k}^{Gait}-H^{Gait}{\hat{x}}_{k|k-1})$$

(26) $$P_{k|k}=(I-K_{k}H^{Gait})P_{k|k-1}$$

To demonstrate the advantages of the proposed EKF fusion method, comparisons were conducted in a simulation environment against UWB-only positioning, gait-only positioning, and a complementary filter fusion method. Fifty Monte Carlo simulation runs were performed in a typical indoor environment measuring 20 × 15 m, the results are presented in Table 5.

The results demonstrate that the proposed EKF fusion method outperforms the other approaches in terms of positioning accuracy, achieving an RMSE of 0.078 m.

To clearly demonstrate the novelty and comprehensive advantages of the proposed method, Table 6 compares the UWB-Gait fusion scheme presented in this work with two mainstream schemes from the literature (UWB + IMU and UWB + RGBD-VO) across key performance dimensions:

In summary, the proposed scheme demonstrates unique advantages in target-specific identification and adaptability to complex scenarios while maintaining high positioning accuracy.

The EKF outputs the optimal state estimate ${\hat{x}}_{k|k}$ at each filtering cycle. The position information (*p_x_*, *p_y_*) is then stored in the trajectory sequence.

**Figure 8:**
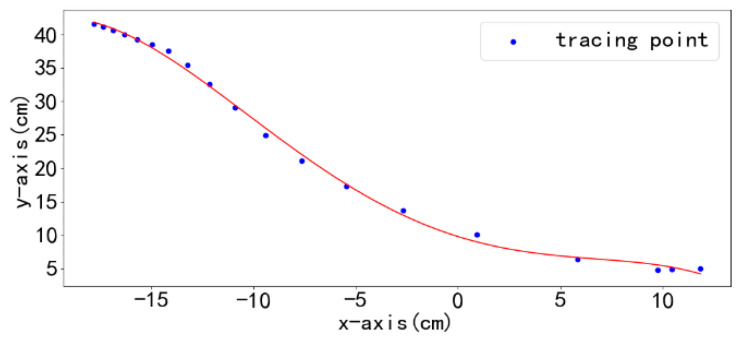
Quadratic polynomial fitting trajectory points. The red line denotes the motion trajectory.

After obtaining the motion trajectory points of the leader, a fourth-order polynomial is employed to fit these points, generating a smooth path as shown in Figure 8. To mitigate cumulative errors, the system resets the origin of the generalized coordinate system every 15 s.

## 5. Design of Car Motion Controller Based on MPC

Following the acquisition of the estimated leader trajectory via the EKF, this chapter aims to design a tracking controller to drive the mobile platform for accurate and smooth trajectory tracking. Given the platform’s kinematic constraints and tracking performance requirements, the Model Predictive Control (MPC) approach is employed in this work [25].

### 5.1. Kinematic Model

The autonomous following vehicle in this study is a differential-drive platform operating at relatively low speeds [26]. Therefore, a two-degree-of-freedom kinematic model suffices [27], with the vehicle subject to the nonholonomic constraint:

(27) $$\dot{x}sin\theta -\dot{y}cos\theta =0$$

**Figure 9:**
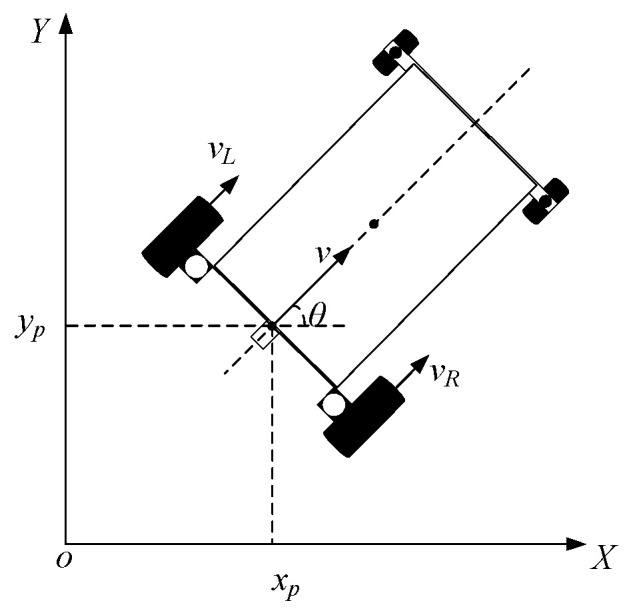
Kinematic model of automatic following car.

The motion model of the mobile platform is illustrated in Figure 9.

A global coordinate system is established with point o in the motion plane as the origin, and the midpoint p between the left and right driving wheels is taken as the reference point of the mobile platform. The state vector is defined as *q = [xp, yp, θ]*, and the control input as *u = [vL, vR]T*, representing the velocities of the left and right wheels, respectively. The platform’s center velocity v and kinematic equations can then be expressed as:

(28) $$v=\frac{v_{L}+v_{R}}{2}$$

Kinematics equation of the vehicle:

(29) $$\left\{\begin{array}{c} \dot{x}=v\cos\theta =\frac{v_{L}+v_{R}}{2}\cos\theta =f_{1}\\ \dot{y}=v\sin\theta =\frac{v_{L}+v_{R}}{2}\sin\theta =f_{2}\\ \dot{\varphi }=\frac{v_{R}-v_{L}}{2d}=f_{3} \end{array}\right.$$

This model constitutes a continuous-time nonlinear system:

(30) $$\dot{\xi }=f(\xi ,u)$$

To apply linear MPC, discretization and first-order Taylor expansion are performed around the reference trajectory point (*q_r_*, *u_r_*), yielding a linear time-varying error model:

(31) $$\tilde{q}\left(k+1\right)=\left[\begin{array}{ccc} 1 & 0 & -\sin\theta_{r}\frac{v_{Lr}+v_{Rr}}{2}\\ 0 & 1 & \cos\theta \frac{v_{Lr}+v_{Rr}}{2}\\ 0 & 0 & 1 \end{array}\right]\tilde{q}\left(k\right)+\left[\begin{array}{cc} T\frac{\cos\theta }{2} & T\frac{\cos\theta }{2}\\ T\frac{\sin\theta }{2} & T\frac{\sin\theta }{2}\\ T\frac{1}{2d} & -T\frac{1}{2d} \end{array}\right]\tilde{u}\left(k\right)=a\tilde{q}(k)+b\tilde{u}(k)$$

(32) $$y(k)=\left[\begin{array}{ccc} 1 & 0 & 0\\ 0 & 1 & 0\\ 0 & 0 & 1 \end{array}\right]\tilde{q}(k)=c\tilde{q}(k)$$

By constructing the augmented state vector:

(33) $$\xi (k)={\left[\;{\tilde{q}\left(k\right)}^{T},{\tilde{u}\left(k-1\right)}^{T}\right]}^{T}$$

and taking the control increments △$\tilde{u}\left(k\right)$ as the new optimization variables, a new state-space equation and output equation suitable for MPC design are derived:

(34) $$\xi \left(k+1\right)=\left[\begin{array}{c} \tilde{q}\left(k+1\right)\\ \tilde{u}\left(k\right) \end{array}\right]=\left[\begin{array}{c} a\tilde{q}\left(k\right)+b\tilde{u}\left(k\right)\\ \tilde{u}\left(k\right) \end{array}\right]=A\xi (k)+B△\tilde{u}(k)$$

(35) η(k)=Cξ(k)

### 5.2. Predictive Model of Controller

Let the prediction time domain be *N_p_* and the control time domain be *N_c_*(*N_p_ ≥ N_c_*). Based on the augmented linear model, the relationship between the predicted system output vector *Y(k)* over the future N_p_ steps and the control increment vector *ΔU(k)* over the future *N_c_* steps can be derived through iterative computation:

(36) Y=ψξ(k)+ΘΔU

The system output of the future time-domain *Nc* can be predicted by knowing the current state and the control increment in the control time-domain *Np*.

### 5.3. Model Prediction and Optimization Solution

The objective of the MPC is to optimize the control increments over the future *N_c_* steps such that the predicted outputs over the future *N_p_* steps closely track the reference output [28,29]. The optimization objective function is defined as:

(37) $$J={\tilde{Y}}^{T}Q_{Q}\tilde{Y}+\Delta U^{T}R_{R}\Delta U=\Delta U^{T}\left(\Theta^{T}Q_{Q}\Theta +R_{R}\right)\Delta U+2E^{T}Q_{Q}\Theta \Delta U+E^{T}Q_{Q}E\;-Y_{r}Q_{Q}\Theta \Delta U+{Y_{r}}^{T}Q_{Q}Y_{r}-2{Y_{r}}^{T}Q_{Q}E$$

where *Q* and *R* are the weighting matrices, and *E(k)* denotes the output error vector. Substituting the prediction model into this expression transforms the objective function into a standard quadratic form.

The optimization problem is subject to the following constraints. Control input constraints: Based on the physical limits of the hub motors, the control input bounds are set as *u_min_* = [−1.5, −1.5]^T^m/s and *u_max_* = [1.5, 1.5]^T^m/s. Control increment constraints: To limit the rate of change of control commands and ensure smooth motion, the control increment bounds are set as *Δu_min_* = [−0.8, −0.8]^T^m/s and *Δu_max_* = [0.8, 0.8]^T^m/s. The MPC parameters in this work are configured as follows: prediction horizon *N_p_* = 20, control horizon *N_c_* = 10, and sampling time *T* = 0.1 s. The error weighting matrix *Q* = diag(10.0, 10.0, 5.0) prioritizes position tracking accuracy, while the control increment weighting matrix *R* = diag(0.1, 0.1) ensures smooth control outputs.

### 5.4. Motion Controller Simulation

**Figure 10:**
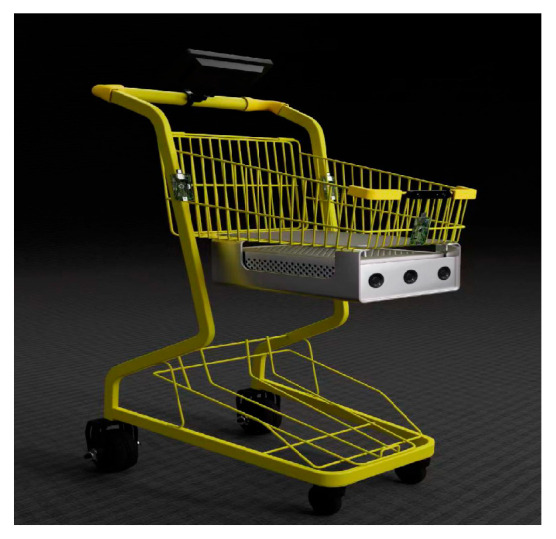
Automatic following car simulation model.

To systematically validate the performance of the tracking controller, a simulation model of the shopping cart mobile platform was constructed using Gazebo 11 under the Ubuntu 20.04 and ROS Noetic framework (Figure 10). The optimization problem of the controller was efficiently solved using the qpOASES solver (Version 3.2.1).

**Figure 11:**
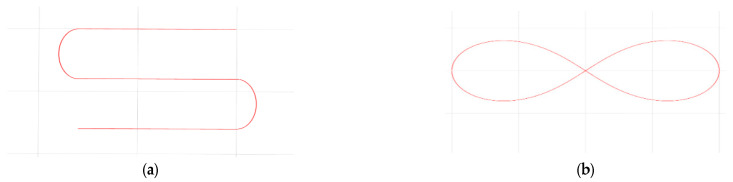
Simulation test track. (**a**) U-shaped trajectory, (**b**) Figure 8 trajectory.

To comprehensively evaluate the controller performance, a U-shaped trajectory (for testing curvature variation response) and a figure-eight trajectory (for testing stability during directional transitions) were designed, as shown in Figure 11. Comparative tests were conducted at two target speeds (1.2 m/s and 1.8 m/s) under both unloaded and loaded conditions (total mass of 23 kg). The physical parameters of the vehicle are provided in Table 7.

**Figure 12:**
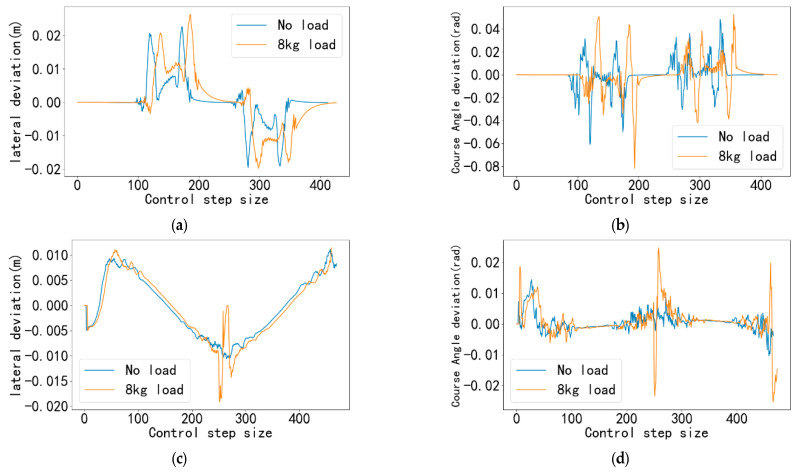
Simulation results at desired speed of 1.2 m/s. (**a**) Lateral deviation of U-shaped trajectory under 1.2 m/s expected velocity (**b**) Course Angle deviation of U-shaped trajectory under 1.2 m/s expected Velocity (**c**) Lateral deviation of figure-8 trajectory under 1.2 m/s expected velocity (**d**) Course Angle deviation of Figure 8 trajectory under 1.2 m/s expected Velocity.

**Figure 13:**
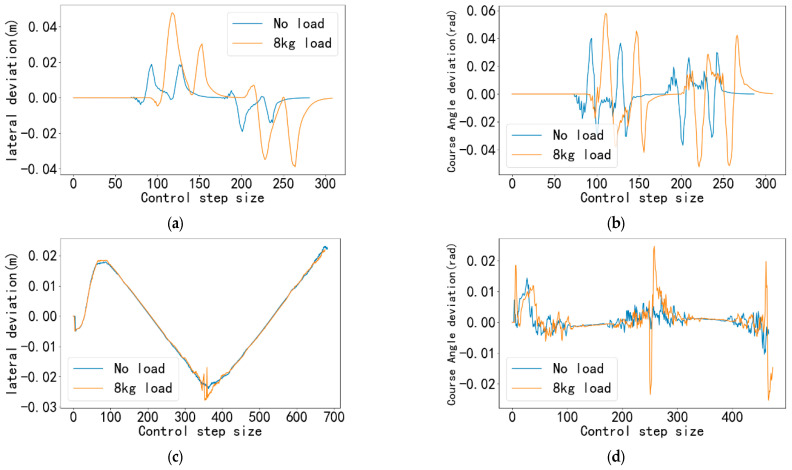
Simulation results at desired speed of 1.8 m/s. (**a**) Lateral deviation of U-shaped trajectory under 1.8 m/s expected velocity (**b**) Course Angle deviation of U-shaped trajectory under 1.8 m/s expected Velocity (**c**) Lateral deviation of figure-8 trajectory under 1.8 m/s expected velocity (**d**) Course Angle deviation of Figure 8 trajectory under 1.8 m/s expected Velocity.

The real-time lateral deviation and heading angle deviation of the car are recorded during the simulation as shown in Figure 12 and Figure 13.

Through the analysis of Figure 12 and Figure 13, the performance of the proposed tracking controller under different paths and velocities can be verified. The results demonstrate that although expected deviation peaks occur at points of sudden curvature change, the controller can quickly stabilize the system, exhibiting favorable convergence and robustness.

For the U-shaped trajectory, as shown in Figure 12a and Figure 13a, the lateral deviation peaks predominantly occur at the four path turning points. This phenomenon is primarily due to the inherent transient error between the predictive model of the model predictive controller and the actual dynamic system. When the path direction changes abruptly, the discrepancy between the future states of the system and the predictions of the model reaches a maximum within a short period, leading to peak tracking deviations. Analysis in Figure 13a,b indicates that higher desired velocities introduce larger peak deviations. This is because, at increased speeds, the vehicle possesses greater kinetic energy, requiring larger control forces to alter its motion direction. Nevertheless, even under demanding conditions such as 1.8 m/s high speed with payload, the maximum lateral deviation remains within 0.05 m. This result fully validates the effectiveness of the controller and meets the precision requirements for indoor mobile platforms operating in confined spaces.

In contrast to the U-shaped trajectory, the figure-eight trajectory exhibits more continuous and gradual curvature variations, avoiding stepwise directional changes. Consequently, the controller can better utilize the predictive capability of MPC to generate smooth control commands in advance, suppressing the maximum lateral deviation to within 0.03 m. This confirms the excellent tracking performance of the controller for continuous-curvature paths. The variation trend of the heading angle deviation (Figure 12b,d and Figure 13b,d) is highly consistent with that of the lateral deviation, with peaks also occurring at points of sudden curvature change. It can be observed that after passing each turning point, both the lateral and heading angle deviations decay rapidly and return to a stable state near zero within a very short time. This demonstrates the high response speed and strong stability of the controller’s feedback correction mechanism, enabling quick suppression of disturbances.

The simulation results indicate that the proposed tracking controller effectively handles the challenges of tracking complex paths such as U-shaped and figure-eight trajectories. The deviations generated at points of sudden curvature change are within reasonable limits, and the controller exhibits fast convergence. Furthermore, parameter analysis shows that increasing the prediction horizon can further enhance performance. In conclusion, the controller provides a high-precision and highly stable solution for trajectory tracking of low-speed differential-drive platforms.

## 6. Hardware Design of the System

**Figure 14:**
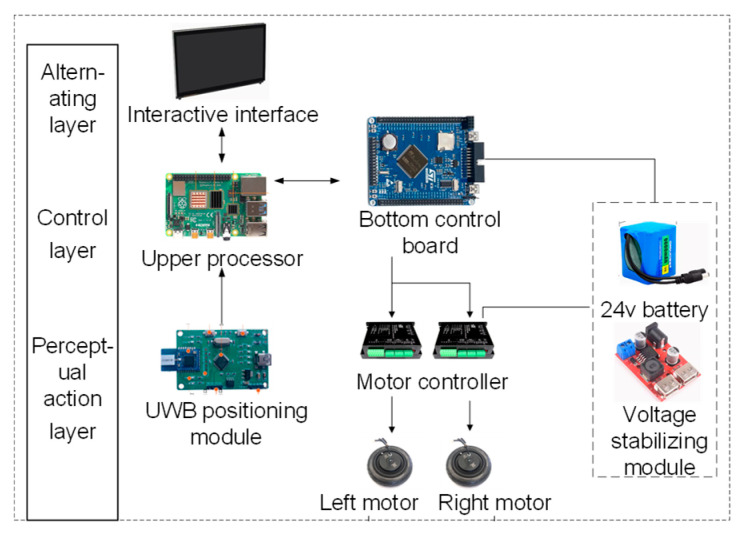
System hardware solution design.

Based on a traditional shopping cart, the following modifications have been made: a camera is integrated at the front of the vehicle for dynamic target detection, local environment perception, and gait feature extraction. Three Ultra-Wideband (UWB) base stations are deployed on the cart body to form a positioning network, enabling real-time computation of the cart’s two-dimensional pose. The front wheel remains as a caster wheel to maintain steering flexibility, while the rear wheels are replaced with dual hub motor-driven electric wheels. This configuration—front-wheel steering and rear-wheel drive—forms a stable “front-steering, rear-drive” architecture. The electronic hardware system of the following cart is divided into an interaction layer, a control layer, and a perception–execution layer. Each layer operates independently, with communication between layers achieved via serial interfaces. A separate power supply layer provides stable electricity to the entire system. The overall hardware architecture is illustrated in Figure 14.

The perception–execution layer includes UWB positioning sensors, an attitude sensor, two motor drivers, and left and right hub motors. This layer is primarily responsible for position sensing and motion execution.

To achieve centimeter-level relative positioning, the UWB positioning module employs the DecaWave DW1000 chip (Decawave Ltd., Dublin, Ireland). Its Time-of-Flight (ToF) ranging principle offers inherent resistance to multipath effects compared to Received Signal Strength Indicator (RSSI)-based solutions. A custom-designed PCB integrated with an STM32F103 minimum system replaces off-the-shelf modules, optimizing SPI communication and data processing to increase the positioning update rate from the conventional 10–20 Hz to over 100 Hz. This provides low-latency data essential for high-speed Model Predictive Control (MPC). To address UWB’s limitations in occluded or target-loss scenarios and to capture rich gait features, a vision sensor is incorporated. The ORBBEC Astra Pro camera (Shenzhen, China) is selected for its active infrared structured light technology, which delivers more stable and reliable depth information under variable indoor lighting conditions compared to passive stereo vision solutions. Its operational range of 0.5 m to 8 m optimally covers typical interpersonal following distances. The camera provides triple data streams—RGB, depth, and infrared—offering extensive flexibility for future vision-based gait recognition algorithms. The motor drivers utilize RS485 communication interfaces, which exhibit strong common-mode noise rejection, making them suitable for long-distance reliable communication in electrically noisy environments. The power system employs an LM2596 switching voltage regulator for its high conversion efficiency (>85%), significantly reducing thermal losses and extending operational endurance compared to linear regulators (LDOs). Two 24 V DC hub motors serve as the motion actuators, each with a rated power of 150 W, a maximum torque of 6 N∙m, and a maximum speed of 1000 RPM (18 km/h).

The control layer consists of an upper-level processor and a lower-level control board. The upper-level processor is a Raspberry Pi 4B (Cambridge, UK), equipped with a quad-core Cortex-A72 processor. It provides sufficient computational power for running a Linux operating system, visual perception algorithms (OpenCV), UWB data fusion, and advanced control strategies such as MPC. With abundant USB and GPIO ports, it easily interfaces with various peripherals. Its responsibilities include: exchanging data with interactive interfaces such as displays; receiving and processing UWB positioning data; bidirectional communication with the lower-level control board; acquiring real-time wheel speed feedback from motor controllers; fusing all sensor data; and issuing motion control commands to the lower-level board. The lower-level control board uses an STM32F103 microcontroller (STMicroelectronics, Geneva, Switzerland), which captures real-time motor speeds via encoder interfaces at high frequencies and communicates reliably with motor drivers over RS485. This dual-computing architecture—Raspberry Pi for decision-making and STM32 for execution—separates non-real-time and real-time tasks, ensuring precise and stable motion control. Key functions implemented include outputting motor control signals and transmitting real-time velocity information of the platform.

The interaction layer comprises a portable low-power display, keyboard, and mouse, providing a visual interface for development and debugging personnel. To meet the power requirements and ensure voltage safety, the power module incorporates 18 lithium batteries (each 500 mAh) connected in three parallel sets of six series (3P6S), forming a battery pack with a nominal voltage of 21.6 V and a capacity of 1500 mAh. This configuration meets both the voltage and capacity demands of the 24 V motor system and various chip-level modules. The LM2596 switching regulator is adopted for its higher power efficiency (typically >80%) compared to linear regulators (e.g., LDO), significantly reducing heat dissipation and extending the system’s continuous operation time.

**Figure 15:**
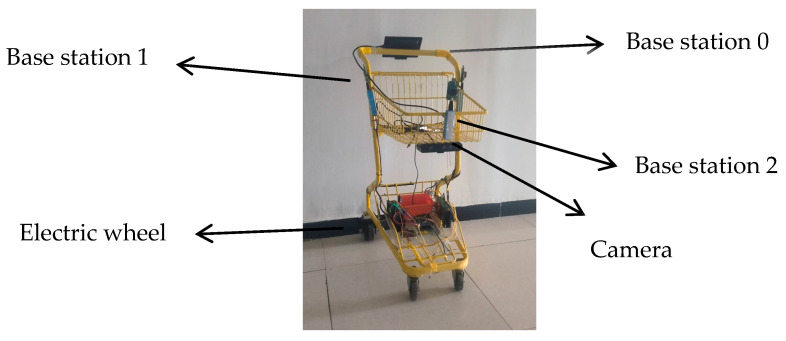
UWB base station sensor layout diagram.

**Figure 16:**
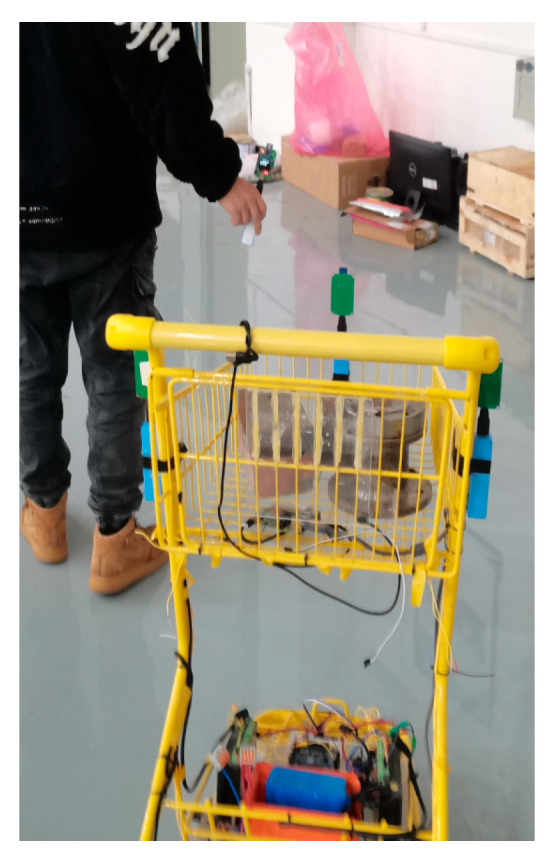
Schematic Depiction of UWB Anchors and the Tracking Target.

Three UWB anchors are deployed on the mobile platform to form an asymmetric positioning network (as shown in Figure 15 and Figure 16). A three-anchor configuration is adopted because it represents the minimum number required for trilateration in a two-dimensional plane while eliminating position ambiguity. The asymmetric layout is chosen to achieve a more favorable geometric arrangement of anchors in the forward direction—the primary direction of movement—thereby improving positioning accuracy along this axis. The leader carries a tag, which optimizes the broadcast timing to communicate with all anchors. Ultimately, all ranging data are aggregated at Anchor 0 and transmitted via a serial interface to a host computer for centralized calculation.

## 7. Car Automatic Following Experiment

### 7.1. UWB Positioning Module Experiments

To validate the practical performance of the UWB relative positioning system, tests were sequentially conducted on ranging accuracy, static positioning accuracy, and positioning accuracy in dynamic environments.

The ranging accuracy between the three base stations and the tag was validated at two typical distances of 200 cm and 600 cm. Multiple measurements were conducted for each base station at each distance, with the results presented in Table 8 and Table 9.

Test results demonstrate that the system achieves a ranging error of less than 3 cm at close range (200 cm), while maintaining the error within 7 cm even at the extended range (600 cm). This level of ranging accuracy adequately meets the requirements for subsequent positioning and following tasks.

A rectangular testing area bounded by the coordinates (−4 m, −2 m), (4 m, −2 m), (−4 m, 8 m), and (4 m, 8 m) was established in the laboratory. The coordinate system was configured with Base Station 2 on the mobile platform as the origin, the platform’s forward direction as the positive Y-axis, and its rightward direction as the positive X-axis. Within this area, 20 sampling points were designated. The UWB tag was used to collect positioning data at these points, which were then compared against the ground truth coordinates to calculate the positioning deviation, with the results presented in Table 10.

The test results indicate an average positioning error of approximately 5 cm in both the X and Y axes. Error analysis reveals that positioning accuracy correlates with the tag-to-base-station distance, with a slight increase in error at greater distances. Since the test area exceeds the operational range of actual following scenarios, the static positioning accuracy of the system fully meets the requirements for low-speed following applications.

Dynamic testing was conducted in an L-shaped corridor to simulate complex indoor environments. Six UWB base stations were deployed to ensure full coverage of the area, while the low-speed mobile platform carried three base stations to establish a local coordinate system. The leader simultaneously carried both the tag of the system under test and a tag of a high-precision reference positioning system. The leader walked along a predetermined path at a walking speed of approximately 1.2 m/s, with both systems synchronously recording trajectory data at 10 Hz.

The localization error at each time step was calculated as follows:

(38) $$error=\sqrt{{\left(x_{est}-x_{gt}\right)}^{2}+{\left(y_{est}-y_{gt}\right)}^{2}}$$

The true distance between the leader and the vehicle at each time step was calculated as:

(39) $$d=\sqrt{{x_{gt}}^{2}+{y_{gt}}^{2}}$$

The true azimuth angle was calculated as:

(40) $$\theta =atan2(y_{gt},x_{gt})$$

**Figure 17:**
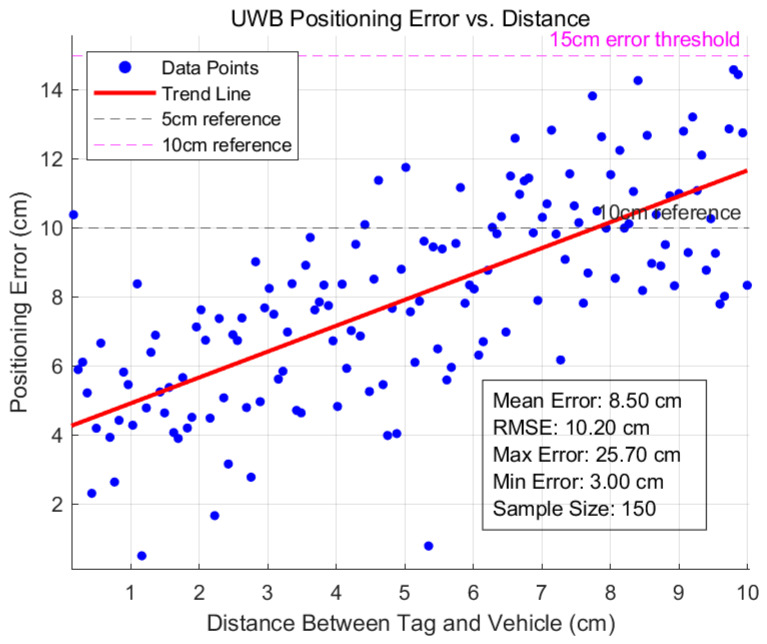
UWB Positioning Error vs. Distance.

The overall error statistics are summarized in Table 11, while the positioning error as a function of the tag-vehicle distance is shown in Figure 17.

The results demonstrate that the positioning errors in dynamic and complex environments are predominantly constrained within 0.15 m, which meets the accuracy requirements for low-speed following applications.

### 7.2. Experiment on the Influence of Mobile Platform Speed on Gait Recognition Accuracy

To evaluate the impact of the mobile platform’s own motion on the performance of visual gait recognition algorithms, this experiment aims to quantitatively analyze the variation patterns of gait recognition accuracy by vehicle-mounted visual sensors under different operating speeds, and verify the necessity of the UWB and gait fusion strategy in dynamic scenarios. The experiment was conducted in a flat indoor corridor with a length of 20 m, ensuring stable lighting conditions. The tracked target walked in a straight line at a speed of approximately 1.2 m/s, maintaining a natural and stable gait. The automatic following trolley followed behind the leader at preset different speeds (0.3 m/s, 0.6 m/s, 0.9 m/s, 1.2 m/s, 1.5 m/s). Data were continuously collected for 60 s at each speed, and the experiments were repeated three times with the results subsequently averaged. The findings are presented in Table 12.

This experiment confirms that gait recognition accuracy progressively decreases as the vehicle’s operational speed increases. A notable degradation in the performance of the visual algorithm is observed particularly when the speed exceeds 1.2 m/s. This finding underscores the necessity of the proposed UWB and gait fusion strategy: during low-speed phases, the system can rely on high-precision visual gait information, whereas at higher speeds, it progressively transitions to greater dependence on the absolute positioning information provided by UWB.

### 7.3. Following Experiment for Low-Speed Mobile Platform

An integrated automatic following experiment was conducted by deploying all systems and the vehicle motion controller onto the experimental vehicle.

**Figure 18:**
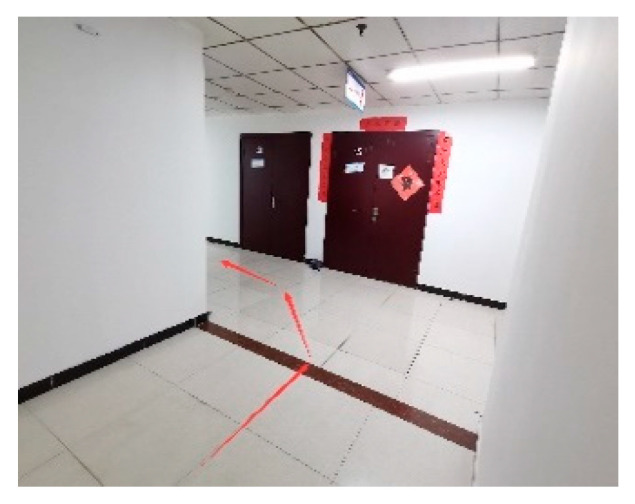
Lab environment.

**Figure 19:**
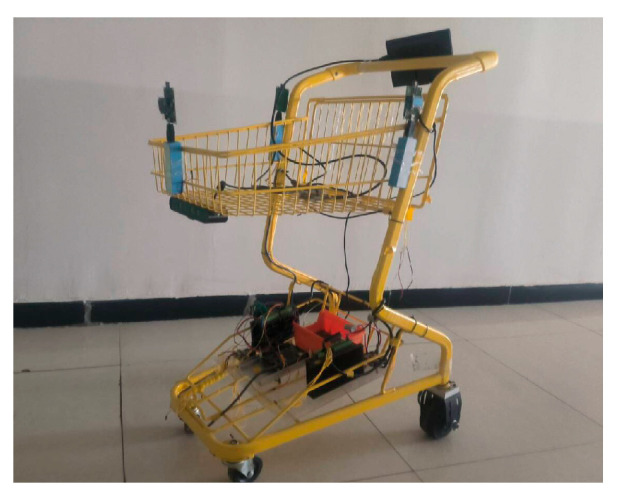
Lab Platform.

**Figure 20:**
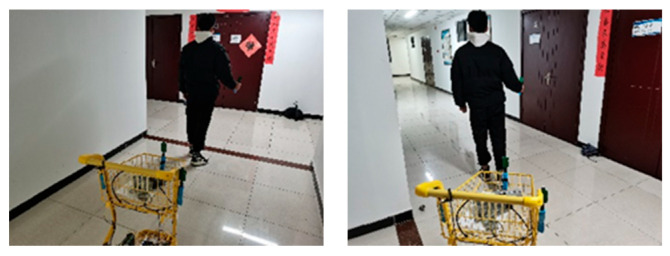
Car following experiment.

The experiment was carried out in a corridor environment (Figure 18) using a shopping cart platform (Figure 19). To accurately record the absolute trajectories of both the leader and the following vehicle for analysis, a UWB-based global reference positioning system was deployed in the corridor environment. In the world coordinate system, the origin was set at the rear corner of the corridor, with the X-axis aligned along the corridor, the Y-axis perpendicular to the corridor, and the Z-axis vertical upward. For data recording, an additional set of positioning base stations was fixed at point (0, 0, 2.6) in the experimental area to serve as the main base station. Both the vehicle and the leader carried a positioning tag for localization within the environment, enabling the recording of their trajectory points. During the experiment, the operator held the UWB positioning tag of the autonomous following system to act as the leader and provide a target for the vehicle to follow. Figure 20 shows the autonomous following experiment in progress, capturing the vehicle following the leader’s movement.

In the course of the experiment, the positioning system demonstrated accuracy in relation to the leader, with no signal loss occurring. Communication across the various system levels was stable, allowing the upper processor to promptly extract both the leader’s movement trajectory and its own attitude orientation through positioning data and real-time wheel speed information. The experimental setup was divided into two conditions: one under a no-load scenario and the other under an 8 kg load condition.

**Figure 21:**
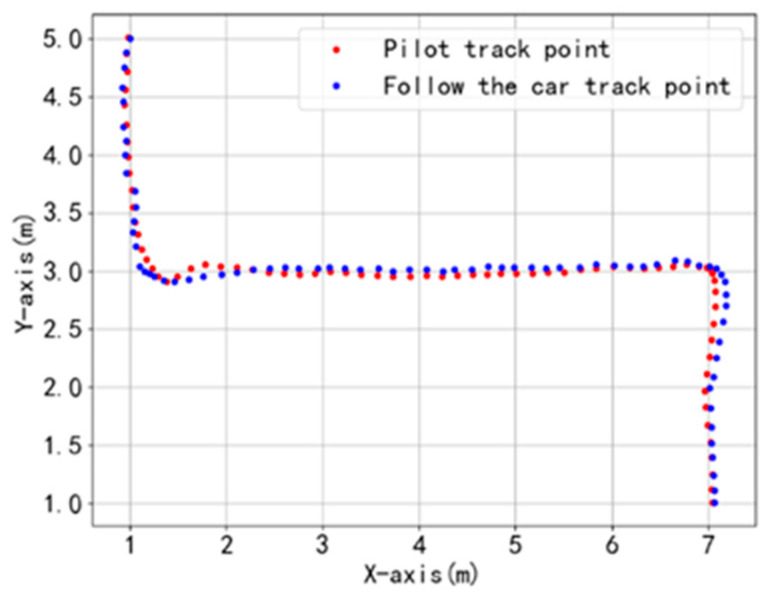
Follow test dynamic trajectory points under no-load conditions.

Under no-load conditions (Figure 21), the system maintains tracking errors within 50 mm during straight-line segments, demonstrating excellent tracking accuracy. The maximum deviation (115 mm) occurs during turns, which is attributed to the transient response delay of the MPC when adjusting differential speeds under abrupt curvature variations. Nevertheless, the controller demonstrates rapid convergence, indicating effective dynamic regulation capability.

**Figure 22:**
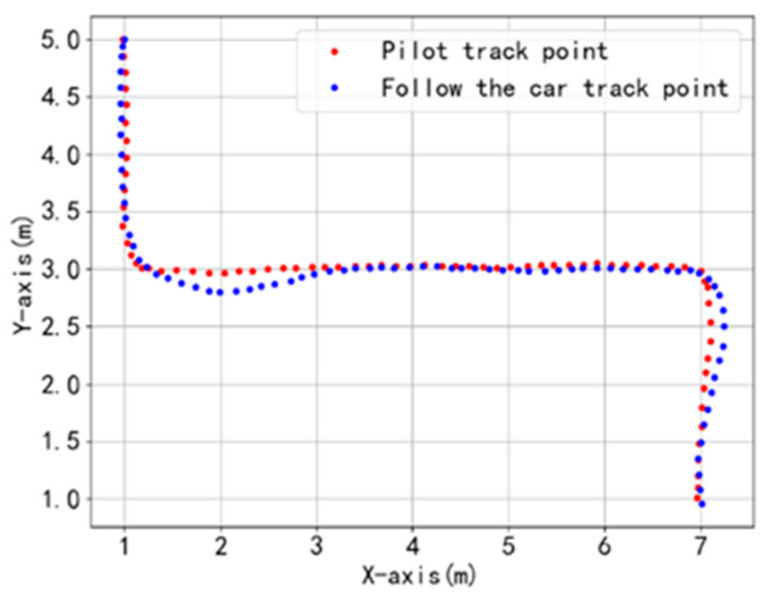
Follow test dynamic trajectory points under 8 kg load conditions.

Under a 15 kg load (Figure 22), the maximum deviation during turns increases to 185 mm, showing a noticeable rise compared to the no-load condition. This is primarily due to the increased rotational inertia of the platform under load, resulting in a slower steering response. However, the tracking accuracy in straight-line segments remains consistent with the no-load case, confirming the MPC’s inherent robustness to mass variations. Further performance optimization could be achieved through online identification of load parameters in future work.

To evaluate the practical deployment feasibility of the system, this section presents a quantitative analysis of the computational load, latency, and overall system power consumption throughout the algorithm pipeline. The tests were performed on Raspberry Pi 4B, which served as the core computing platform, without utilizing an external GPU. The testing scenario involved the system operating in a standard following state with the leader walking normally.

During stable system operation, system resources were monitored using the Linux top command for CPU utilization and the rpi-gpumon tool for GPU utilization, with additional hardware data from vcgencmd, the results are summarized in Table 13.

Analysis indicates that the system’s computing resources are relatively tight, with the visual perception modules (YOLOv5 and GaitPart) being the primary source of the computational burden, as their GPU utilization has reached the limit. Future work will focus on optimization through model lightweighting or utilizing more powerful hardware platforms.

Using high-precision timers, the processing latency of key perception stages was measured. The results represent the average value obtained from 1000 consecutive measurements, the results are summarized in Table 14.

The total latency from perception to control is approximately 210 ms. Given the relatively low speed (about 1.2 m/s) of the mobile platform relative to the leader, this latency is within a manageable range but remains one of the factors contributing to tracking deviation. The execution rate (10 Hz) of the MPC meets the requirements for real-time control.

The total system power consumption (including the compute unit, sensors, and motor standby power) was measured using a power meter under both the unloaded standby and typical tracking operational states, the results are summarized in Table 15.

Power consumption is primarily concentrated in the mechanical work of driving the motors, with the computing unit itself contributing a relatively minor share.

In summary, the current system on its existing hardware platform meets the basic requirements for real-time following, although the computing resources are nearing saturation. The performance metrics outlined above provide a clear basis for subsequent system optimization and product design.

## 8. Conclusions

This paper successfully designed and implemented an automatic following system for low-speed mobile platforms that integrates UWB positioning and visual gait recognition. The experimental results demonstrate the system’s excellent performance in indoor environments, with core achievements reflected in the following three aspects.

Firstly, at the perception level, the EKF fusion strategy adopted in this paper proved highly effective. It not only achieved a localization accuracy (RMSE 0.078 m) superior to any single sensor, but more importantly, it enabled effective fault tolerance against non-line-of-sight (NLOS) conditions through the cross-modal complementarity of UWB and gait information. The core mechanism lies in the EKF’s ability to autonomously reduce the weight of UWB data when anomalies are detected, relying instead on the continuous motion estimates from visual gait tracking to maintain stable following. This capability was indirectly validated by field tests where the system avoided losing track in complex corridors; simulation analysis further indicated tolerance to UWB signal outages lasting several seconds. This fusion strategy establishes a reliable perceptual foundation for the entire system.

Secondly, regarding control and overall performance, the tracking controller based on Model Predictive Control (MPC) effectively handled the platform’s kinematic constraints, achieving smooth and stable trajectory tracking. Field tests confirmed the system’s practicality: in typical indoor environments, the average tracking error was less than 50 mm, with the maximum error not exceeding 185 mm, fully demonstrating its ability to accurately understand the leader’s intent and achieve reliable autonomous following.

Nevertheless, this study has limitations. Experiments were primarily conducted in controlled scenarios with a single leader. The system’s adaptability in multi-person interactions, under severe lighting variations, and for dynamic obstacle avoidance in unstructured environments requires further enhancement. Additionally, the MPC does not account for the dynamic effects of load variations, and the system’s real-time performance offers room for further optimization. Precisely quantifying the fault tolerance thresholds for conditions like NLOS is also a key focus for future research

This research provides a feasible technical solution and solid theoretical support for the practical application of intelligent mobile platforms in high-frequency human–robot collaboration scenarios such as logistics handling and medical transfer.

## Figures and Tables

**Table 1:** UWB Tag Endurance Test Using Traditional Ranging Sequence.

Metric	Single-Base Station Mode	Dual-Base Station Mode	Three-Base Station Mode	Four-Base Station Mode
Number of Signal Transmissions (Tag)	2	4	6	8
Battery Life (min, with a 1500 mAh battery)	68	52	43	37

**Table 2:** UWB Tag Endurance Test Using Improved Ranging Sequence.

Metric	Single-Base Station Mode	Dual-Base Station Mode	Three-Base Station Mode	Four-Base Station Mode
Number of Signal Transmissions (Tag)	2	2	2	2
Battery Life (min, with a 1500 mAh battery)	65	63	61	62

**Table 3:** Recognition Accuracy (%) of Different Methods on the CASIA-B Dataset.

Metric	Normal Walking (NM)	Carrying a Bag (BG)	Wearing a Coat (CL)	Average
GEI + SVM	89.1	75.4	64.3	76.6
HOG + RNN	92.3	80.1	69.5	80.7
The method in this paper	96.8	91.2	83.5	90.5

**Table 4:** Recognition Robustness under Noise Interference.

Noise Level (σ)	GEI + SVM	HOG + RNN	Proposed Method
σ = 0.05	88.5%	93.5%	95.8%
σ = 0.10	82.1%	88.7%	92.4%
σ = 0.20	69.3%	78.3%	85.9%

**Table 5:** Positioning error comparison of different methods (Unit: m).

Method	RMSE-X	RMSE-Y	RMSE-Total	RMSE-Max
UWB-only	0.083	0.081	0.123	0.353
Gait-only	0.152	0.168	0.226	0.893
Comp Filter	0.071	0.075	0.108	0.283
The method in this paper	0.035	0.038	0.049	0.112

**Table 6:** Comparison of UWB-Based Multi-Sensor Fusion Schemes.

Comparison Dimension	UWB + IMU	UWB + RGBD-VO	Proposed Scheme
Core Positioning Accuracy	Medium (subject to IMU drift)	Medium-High (highly environment-dependent)	High
Target Identification	None (geometric positioning only)	Limited (relies on appearance features)	Strong (based on gait features)
UWB NLOS Robustness	Poor (rapid IMU drift)	Medium (visual-assisted NLOS suppression)	Superior (low-drift estimation)
Visual Occlusion Robustness	Excellent (vision-independent)	Poor (fails under occlusion)	Good (can degrade to UWB-only mode)
Technical Bottleneck	No target identification; IMU drift	environment-sensitive; appearance ambiguity	motion blur susceptibility

**Table 7:** Car simulation parameters.

Parameter Name	Parameter Value
Driving wheel radius (m)	0.06
Driving wheel spacing (m)	0.38
Total mass at no load (kg)	15
Total mass under load (kg)	23
Moment of inertia of driving wheel (kg∙m^2^)	0.0014
Height of centroid under no load (m)	0.45
Centroid height under load (m)	0.54
Distance from the center of mass to the rear axis(m)	0.18

**Table 8:** Ranging Experiment at Actual Distance of 200 cm.

Base Station ID	1st Ranging Value (cm)	2nd Ranging Value (cm)	3rd Ranging Value (cm)	Average Value (cm)	Mean Error (cm)
Base Station 0	201.6	202.5	202.1	202.1	2.07
Base Station 1	204.9	201.4	197.7	201.3	2.87
Base Station 2	205.4	200.2	203.6	203.1	3.06

**Table 9:** Ranging Experiment at Actual Distance of 600 cm.

Base Station ID	1st Ranging Value (cm)	2nd Ranging Value (cm)	3rd Ranging Value (cm)	Average Value (cm)	Mean Error (cm)
Base Station 0	601.1	601.7	598.9	600.6	1.3
Base Station 1	605.3	611.8	603.5	606.9	6.8
Base Station 2	598.4	599.2	609.9	602.5	4.1

**Table 10:** Positioning Test Deviation Results.

	Max (cm)	Min (cm)	Mean (cm)
X-axis	15.2	1.8	4.8
Y-axis	14.6	1.6	5.2
Overall Deviation	17.6	2.1	5.6

**Table 11:** Summary of UWB Positioning System Error Metrics.

Metric	Value (cm)
Mean Error	8.5
Root Mean Square Error (RMSE)	10.2
Maximum Error	25.7

**Table 12:** Gait Recognition Performance at Different Vehicle Speeds.

Vehicle Speed (m/s)	Step Frequency Error (Steps/min)	Gait Cycle Std (s)	Feature Similarity (%)
0.3	0.8	0.05	96.5
0.6	1.2	0.07	94.2
0.9	2.1	0.12	90.1
1.2	3.5	0.18	85.3
1.5	5.8	0.26	78.6
1.8	9.7	0.41	68.5

**Table 13:** Computational Load of Each Algorithm Module.

Processing Module	CPU Utilization (%)	GPU Utilization (%)
UWB Data Decoding and Filtering	8%	-
YOLOv5 Object Detection	45%	65%
Gait Contour Extraction and GaitPart Inference	25%	35%
EKF Fusion and MPC Solving	15%	-
System Background Processes	10%	-
Total	95%	100%

**Table 14:** Latency of Key Processing Stages.

Processing Stage	Average Latency (ms)	Standard Deviation (ms)
Image Acquisition and Preprocessing	15	2
YOLOv5 Object Detection	120	8
Gait Feature Extraction	65	5
EKF Fusion and MPC Solving	10	1
Total Perception Layer Latency	210	10
Control Command Cycle	100	-

**Table 15:** System Power Consumption.

Operating State	CAverage Power Consumption (W)
System Standby	8 W
System Standby	35 W
Peak Power Consumption	55 W

## Data Availability

No new data were created or analyzed in this study. Data sharing is not applicable to this article.
